# Regulation of histamine and diamine oxidase in patients undergoing orthotopic liver transplantation

**DOI:** 10.1038/s41598-020-57728-x

**Published:** 2020-01-21

**Authors:** Judith Schiefer, Joanna Baron-Stefaniak, Thomas Boehm, Patricia Wadowski, Gabriela Berlakovich, Lorenz Kuessel, Jakob Mühlbacher, Petra Jilma-Stohlawetz, Michael Schwameis, Bernd Jilma, Peter Faybik

**Affiliations:** 1 Department of Anesthesia, General Intensive Care and Pain Management, Medical University of Vienna, Waehringer Guertel 18-20, 1090 Vienna, Austria; 2 Department of Clinical Pharmacology, Medical University of Vienna, Waehringer Guertel 18-20, 1090 Vienna, Austria; 3 Department of Surgery, Division of Transplantation, Medical University of Vienna, Waehringer Guertel 18-20, 1090 Vienna, Austria; 4 Department of Obstetrics and Gynecology, Medical University of Vienna, Waehringer Guertel 18-20, 1090 Vienna, Austria; 5 Department of Laboratory Medicine, Division of Medical and Chemical Laboratory Diagnostics, Medical University of Vienna, Waehringer Guertel 18-20, 1090 Vienna, Austria; 6 Department of Emergency Medicine, Medical University of Vienna, Waehringer Guertel 18-20, 1090 Vienna, Austria

**Keywords:** Liver cirrhosis, Translational research

## Abstract

Increased concentrations of the vasodilator histamine have been observed in patients undergoing abdominal surgery. The role of histamine during orthotopic liver transplantation (OLT) has only been studied in animals. The aim of this study was to measure plasma concentrations of histamine and its degrading enzyme diamine oxidase (DAO) in patients undergoing orthotopic liver transplantation, and assess whether histamine or DAO correlate with intraoperative noradrenaline requirements. Histamine and DAO concentrations were measured in 22 adults undergoing liver transplantation and 22 healthy adults. Furthermore, norepinephrine requirements during liver transplantation were recorded. Baseline concentrations of histamine and DAO were greater in patients, who underwent liver transplantation, than in healthy individuals (Histamine: 6.4 nM, IQR[2.9–11.7] versus 4.3 nM, IQR[3.7–7.1], p = 0.029; DAO: 2.0 ng/mL, IQR[1.5–4.1] versus <0,5 ng/mL, IQR[<0.5–1.1], p < 0.001). During liver transplantation, histamine concentrations decreased to 1.8 nM, IQR[0.5–4.9] in the anhepatic phase (p < 0.0001 versus baseline), and to 1.5 nM, IQR[0.5–2.9] after reperfusion (p < 0.0001 versus baseline). In contrast, DAO concentrations increased to 35.5 ng/ml, IQR[20–50] in the anhepatic phase (p = 0.001 versus baseline) and to 39.5 ng/ml, IQR[23–64] after reperfusion (p = 0.001 versus baseline), correlating inversely with histamine. Norepinephrine requirements during human liver transplantation correlated significantly with DAO concentrations in the anhepatic phase (r = 0.58, p = 0.011) and after reperfusion (r = 0.56; p = 0.022). In patients undergoing orthotopic liver transplantation, histamine concentrations decrease whereas DAO concentrations increase manifold. Diamine oxidase correlates with intraoperative norepinephrine requirements in patients undergoing OLT.

## Introduction

Orthotopic liver transplantation (OLT) is the only curative procedure for end-stage liver disease (ESLD). Patients with ESLD usually present with a systemic hyperdynamic cardiovascular regulation, demonstrating an increased cardiac output, reduced systemic vascular resistance and impaired vascular responsiveness to stress. During OLT, patients are exposed to further major hemodynamic alterations primarily due to temporary caval clamping, blood loss and ischemia/reperfusion (I/R) injury. Temporary clamping of the inferior vena cava decreases cardiac output by approximately 50% due to a reduction in venous return to the heart^1,2^, while blood loss leads to hypovolemia. I/R injury, mainly characterized by oxidative damage and a severe inflammatory response, triggers the secretion of vasoactive mediators into the recipients’ systemic circulation^3^. Thus, OLT is frequently associated with hemodynamic instability of the recipient presenting clinically with hypotension, increased vasopressor support and cardiac arrhythmias.

Histamine is stored primarily in basophils and mast cells, and its release is triggered by various antigens, cytokines and drugs, but also by hypoxia and I/R^4^. When released, histamine exerts pleiotropic physiological and pathophysiological regulatory functions, mainly inducing systemic vasodilation and increasing capillary permeability^3^. Experimental studies have demonstrated that histamine concentrations increase in pigs undergoing OLT^5^. In clinical studies, increased plasma histamine concentrations have been observed in patients with cirrhosis^6^and in patients undergoing abdominal surgeries^7^. However, no human studies have assessed histamine concentrations in patients undergoing OLT.

Balance between release and clearance of histamine is of utmost importance for hemodynamic stability in humans. In order to prevent or minimize an overshooting systemic vasoactive response to histamine, the body is equipped with various defense mechanisms to tightly control the plasma histamine concentrations^6^. Excessive enteral and likely renal re-absorption of histamine are prevented by the constitutive expression of the histamine metabolizing enzyme diamine oxidase (DAO)^8^. Furthermore, the liver is a major organ responsible for clearing excessive histamine from the circulation^9^.

During OLT patients are exposed to an anhepatic period. We hypothesized that in patients undergoing OLT the anhepatic phase induces a dysbalance between release and clearance of histamine, contributing to systemic hypotension during OLT. Thus, the aim of this study was to measure concentrations of histamine and its degrading enzyme DAO in patients undergoing OLT, and to correlate histamine and DAO concentrations with the intra-operative noradrenaline requirements.

## Materials and Methods

### Study subjects

This single-center, observational study was performed in accordance with the ethical standards laid down in the Declaration of Helsinki. Institutional ethics committee (Medical University of Vienna) approval for the study was obtained. Written informed consent was obtained before enrollment into the study.

Twenty-two adult patients scheduled for OLT at the General Hospital of Vienna between August 2014 and August 2015 were enrolled in the study. Exclusion criteria were combined liver-kidney or liver-lung transplantation, and the need of intraoperative veno-venous bypass^10^. To allow direct comparison between patients with ESLD and healthy individuals, blood samples were additionally obtained form twenty-two healthy age-matched adult volunteers.

### Anesthesia, surgery, and immunosuppression

Anesthesia and surgery were performed according to the local standards, which have already been previously described by our research group^10,11^. The surgical technique was performed pursuant to the local standard technique with cross clamping of the inferior caval vein. All grafts were ABO compatible and matched for the graft-to-recipient-weight ratio. Immunosuppression was initiated already intraoperatively, before graft reperfusion, and continued according to the local standardized protocol for immunosuppression in OLT.

### Data and sample collection

Demographic data, primary disease leading to ESLD, the model of end-stage liver disease (MELD) score, surgical reports, the anesthetic report, as well as relevant parameters from the postoperative course on the intensive care unit, including the use of blood and coagulation products were collected for each patient. Diagnosis of clinically significant portal hypertension was established in patients suffering from ESLD fulfilling one or more of the following: hepatic vein pressure gradient >10 mmHg, gastroenteral varices, liver stiffness measurement or imaging showing collateral circulation^12,13^.

Results from routinely daily measured laboratory parameters were extracted from the patient data management software (PDMS, Phillips, USA). The maximal norepinephrine infusion rate and the total amount of infused norepinephrine during OLT were recorded. In patients scheduled for OLT, blood samples were drawn from an arterial line, which is placed standardly during OLT. Arterial blood samples were collected at the following pre-defined time points (TP): at baseline (BL) prior to surgery, during OLT in the anhepatic phase immediately prior to reperfusion (TP1) and 10 minutes after graft - reperfusion (TP2). Control blood samples were obtained in the morning from the radial artery of fasting adult volunteers. Immediately after collection, plasma samples were centrifuged at 2600 g for 20 minutes and stored at **−**80 °C until analysis.

### Sample analysis

Histamine concentrations in plasma were measured using a commercially available enzyme-linked immunosorbent assay (ELISA) (Immunotech, Beckmann Coulter Company, Brea, CA) on a standard curve ranging from 1–90 nM.

Diamine oxidase concentrations were measured using a custom-assembled ELISA as described recently^14^ using recombinant human diamine oxidase as a standard^15^. Briefly, a purified monoclonal antibody raised against human DAO isolated from Caco-2 cell supernatant was coated at 5 µg/ml onto white high protein binding microtiter plates (Greiner bio-one 655074) in 50 mM carbonate-bicarbonate coating buffer pH 9.6 overnight at 4 °C. EDTA plasma was diluted 1 to 5 with LowCross-Buffer (CANDOR bioscience 1000500; Germany). Bound human DAO was detected with an anti-DAO polyclonal rabbit IgG serum fraction diluted 1 to 1000. The bound rabbit antibodies were detected with a 1 to 32000 dilution of donkey anti-rabbit IgG HRP-labelled antibodies (Sigma SAB3700928; Austria). Antibodies were incubated for 50 minutes at room temperature. Bound HRP molecules were quantified with the SuperSignal™ ELISA Pico Chemiluminescent Substrate (Thermo Scientific 37070; Vienna) using a standard chemiluminescent reader (Victor2^TM^ 1420 Microtiter Plate Reader (Perkin Elmer). The limit of blank (LOB) and detection (LOD) are 0.27 and 0.48 ng/ml respectively using 42 standard curve determinations. The estimate limit of quantification (eLOQ) is 0.70 ng/ml. A value of 0.48 ng/mL was assigned for values below the detection limit for conservative statistical comparisons.

Mast cell degranulation triggered by anaphylactic reactions is a frequent cause for histamine release into patient’s systemic circulation^16^, but may also be associated with DAO release^17^. To exclude possible intraoperative mast cell degranulation as a potential cause for histamine release in our patients, total tryptase concentrations were measured in plasma of patients undergoing OLT (ImmunoCAP Tryptase ELISA Kit, Thermoscientific, Waltham, MA).

Diamine oxidase release is triggered by heparin^18^. To determine whether endogenous hepariniziation could explain a possible DAO release, we measured aPTT and anti-FXa activities in a subgroup of patients, and performed a thrombelastometric analysis calculating the difference between measurements with and without heparinase (INTEM – HEPTEM).

### Statistical analysis

Statistical analyses were performed using Prism 6.0 (GraphPad Software, La Jolla, CA) and Statistica, (Stat Soft Inc., Tulsa, OK). Results are depicted as median with interquartile ranges (IQR, [25–75%], unless indicated otherwise. A Kolmogorov-Smirnov test was used to verify normal distribution. A Friedman analysis of variance (ANOVA), followed by post-hoc Wilcoxon tests, and U-tests were applied for comparison between groups. Correlations were calculated by the Spearman ranks correlation analysis. Receiver operating characteristic (ROC) curve was used to assess the diagnostic value of plasma histamine and DAO concentrations to discriminate the need for higher dosage of vasopressor support with norepinephrine (≥0.3 mcg/kg/min) during the anhepatic phase and reperfusion period. Statistical significance was set at *p* < 0.05.

### Sample size calculation

Previous studies assessed plasma histamine concentrations in healthy volunteers^19^, and compared histamine concentrations between cirrhotic patients and healthy individuals^20^. Based on these data we estimated an intra- and inter-subject coefficient of variation of plasma histamine concentrations of approximately 60% and 90%, respectively. We calculated that 19 subjects in each group would be sufficient to allow detection of a 2-fold greater histamine concentration in patients undergoing OLT with 80% power and an alpha error of <5%. We felt that this effect size is clinically justified because plasma histamine concentrations greater than 2-fold the upper normal limit of 11 nM might be hemodynamically relevant as demonstrated by a 30% increase in the heart rate in healthy volunteers^3^.

### Ethics declaration

Approval was obtained from the local ethics committee of the Medical University of Vienna. Patients informed consent was obtained before enrollment in the study.

## Results

### Demographic data

Twenty-one patients (mean age 56 ± 12 years) with ESLD undergoing OLT were enrolled in the study. In addition, one patient with acute liver failure was enrolled. Furthermore, we included 22 healthy age-matched volunteers. Table 1 shows the demographic data and etiology leading to OLT. Table 2 depicts baseline and postoperative laboratory parameters after OLT. Table 3 shows the intraoperative substitution of blood products and crystalloids.

**Table 1:** Demographic data and indications of patients undergoing liver transplantation.

Age (years)	57 (29–72)
Sex (female/male), n	7/15
MELD score	17 (9–40)
Child Pugh Score, n (A/B/C)	(1/8/13)
**Etiology of liver disease**	**N (%)**
Alcohol	7 (31%)
Hepatocellular carcinoma	3 (13%)
Alcohol and hepatocellular carcinoma	1 (5%)
Alcohol and viral	1 (5%)
Viral and hepatocellular carcinoma	2 (9%)
Cholestatic liver disease	2 (9%)
Cryptogenic cirrhosis	2 (9%)
Autoimmune cirrhosis	2 (9%)
Acute Liver Failure	1 (5%)
Re-OLT	1 (5%)
**Comorbidities**	**N (%)**
Diabetes mellitus	7 (32%)
Cardiovascular	8 (36%)
Chronic kidney disease	3 (14%)

Categorical variables are described by absolute and relative frequencies or median and IQR, if applicable. Abbreviations: IQR, interquartile range; MELD, model for end-stage liver disease.

**Table 2:** Perioperative laboratory parameters.

Parameter	Pre-OLT	Post-OLT	Day 1 post OLT
Hemoglobin, (g/dL)	10.8 (7.4–15.3)	9.0 (7.5–11.3)	8.8 (5.7–12)
White blood cells, (10^9^/L)	3.7 (1.3–7.3)	7.9 (0.7–17.2)	8.2 (1.1–34.2)
Platelets, (10^9^/L)	63 (16–239)	47 (21–183)	52 (18–185)
AST, (U/L)	46 (21–336)	1085 (398–10430)	557 (91–16620)
ALT, (U/L)	31 (16–229)	939 (384–3961)	784 (266–8100)
Bilirubin, (mg/dL)	2.9 (0.5–41.7)	4.5 (1–19.3)	3.6 (0.8–20.1)
Creatinine, (mg/dL)	0.8 (0.4–4.4)	1.4 (0.6–5)	1.7 (0.7–5.8)
Prothrombin time, (%)	41 (5–89)	43 (24–129)	47 (14–88)
aPTT, (s)	42 (35–97)	91 (44–173)	46 (36–68)

Data are given as median and IQR. Abbreviations: ALT, alanine aminotransferase; aPTT, activated partial thromboplastin time; AST, aspartate aminotransferase; IQR, interquartile range; OLT, orthotopic liver transplantation.

**Table 3:** Intraoperative substitution of blood products and crystalloids.

Blood products given	
Packed red blood cells, (units)	3 (0–16)
Fresh frozen plasma, (units)	6 (0–20)
Platelets, (units)	1 (0–4)
Crystalloids, (ml)	5625 (2500–9000)
Colloids, (ml)	315 (0–600)
Prothrombin complex concentrates, (units)	1500 (0–6500)

Data are given as median and IQR. Abbreviations: IQR, interquartile range.

### Perioperative plasma histamine concentrations

**Figure 1:**
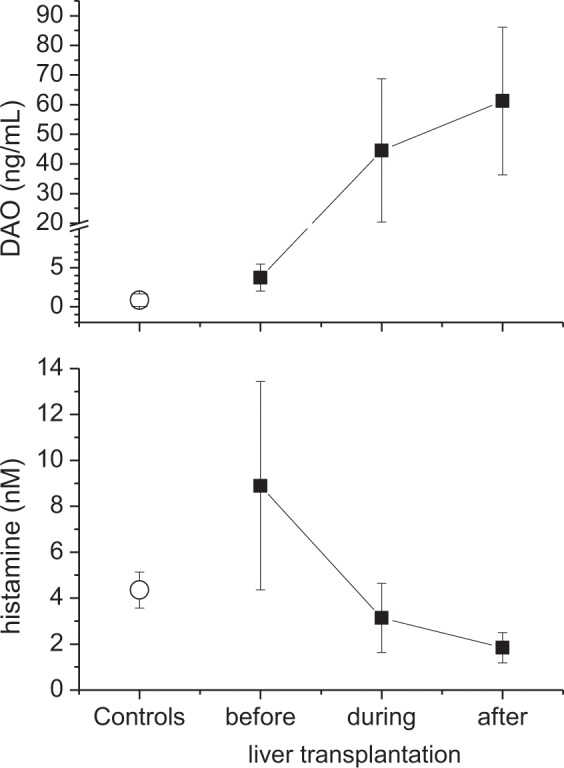
Median plasma concentrations of histamine and DAO. Concentrations of (**A**) plasma histamine and (**B**) plasma DAO in healthy controls, and patients undergoing OLT at 3 different time points: baseline (BL), in the anhepatic phase (TP1), and after reperfusion (TP2).

Median histamine concentrations at BL in all patients scheduled for OLT were significant greater than those in healthy control individuals (6.4 nM, IQR [2.9–11.7] vs. 4.3 nM, IQR [3.7–7.1]; p = 0.029). In patients undergoing OLT, plasma histamine concentrations decreased to 1.8 nM, IQR [0.5–4.9] at TP1 in the anhepatic phase (p < 0.001 versus BL) and to 1.5 nM, IQR [0.5–2.9] after reperfusion (p < 0.001 versus BL; Fig. 1). In 21 patients with ESLD undergoing OLT median plasma histamine concentrations decreased from 6.6 nM (IQR 3.3–11.8) at BL to 1.88 nM (IQR 0.5–5.1) during anhepatic phase (p < 0.001 versus BL) and further to 1.82 nM (IQR 0.5–2.9) after reperfusion (p < 0.001 versus BL). In the only 1 patient with acute liver failure undergoing OLT, plasma histamine concentrations decreased from 2.1 nM at BL to 0.6 nM during the anhepatic phase and to 0.5 nM after reperfusion. Interestingly, histamine concentrations measured after reperfusion were lower in patients undergoing OLT than histamine concentrations of healthy volunteers at BL. Neither the amount and type of fluids, nor the number of blood products administered during transplantation correlated with plasma histamine concentrations.

### Perioperative plasma DAO concentrations

In patients scheduled for OLT, baseline plasma DAO concentrations were significantly greater (2.0 ng/mL, IQR [1.5–4.1]) than in age-matched controls (<0.50 ng/mL, IQR [<0.5 to 1.1]; p < 0.001). In patients undergoing OLT, DAO concentrations increased to 35.5 ng/mL, IQR [20–50] in the anhepatic phase (p = 0.001 versus baseline), and to 39.5 ng/mL, IQR [23–64] in the reperfusion period (p = 0.001 versus baseline) (Fig. 1). Of note, DAO concentrations correlated inversely with histamine concentrations in the anhepatic phase (r = −0,68; p = 0.001) and during reperfusion (r = −0.53, p = 0.02). In patients with ESLD undergoing OLT, median DAO concentrations increased from 2.1 ng/mL (IQR 1.5–4.0) at BL to 36.2 ng/mL (IQR 20.4–50.2) in the anhepatic phase (p = 0.001 versus baseline), and further to 41.6 ng/mL (IQR 24.6–64.3) in the reperfusion period (p = 0.001 versus baseline). In one patient with acute liver failure undergoing OLT, DAO concentrations increased from 17.5 ng/ml at BL to 20.4 ng/mL in the anhepatic phase, and later decreased to 12.6 ng/mL in the reperfusion period. Neither the amount and type of fluids, nor the number of blood products administered during transplantation correlated with DAO concentrations.

### Effect of liver function on plasma histamine and DAO concentrations

There was no statistically significant correlation between MELD score, prothrombin time, serum albumin or total bilirubin with plasma histamine or DAO concentrations at baseline. Furthermore, no significant correlation was found between the plasma histamine or DAO concentrations and AST as a marker of I/R injury at the end of OLT or on day 1 after transplantation.

### Effect of clinical significant portal hypertension on plasma histamine and DAO concentrations

Seventeen patients (80%) with ESLD included in our study fulfilled the criteria of clinically significant portal hypertension, 4 (20%) patients did not. Plasma histamine (p = 0.6) and DAO (p = 0.33) concentration were not significantly different between patients with or without clinical significant portal hypertension at baseline before OLT. The median plasma histamine concentration during reperfusion was significantly higher in patients with clinical significant portal hypertension than in patients without (2.4 nM, IQR [0.5–3.1] vs. 0.54 nM, IQR [0.5–0.5]; p = 0.03). However, there were no statistically significant differences in plasma histamine or DAO concentrations at any other time-point between patients with or without signs of clinical significant portal hypertension during our study.

### Norepinephrine requirements and concentrations of histamine and DAO during OLT

During OLT, the median norepinephrine infusion rate was 0.287 mcg/kg/min, IQR [0.1–1.0]. The median cumulative norepinephrine dose administered during OLT was 4.5 mg [range 1.3–20.0]. There was no significant correlation between plasma histamine concentrations and norepinephrine requirements. Interestingly, the maximal intraoperative norepinephrine infusion rate correlated significantly with DAO concentrations in the anhepatic phase (r = 0.58; p = 0.011) and after reperfusion (r = 0.56; p = 0.022). The total intra-operative norepinephrine dose correlated significantly with DAO concentrations in the anhepatic phase (r = 0.46; p = 0.049), but not in the reperfusion phase (r = 0.38; p = 0.11). In the ROC curve analysis, DAO during the anhepatic phase exhibited an AUC of 0.85 (95% confidence interval, 0.62–0.97; p = 0.0001) for the norepinephrine requirement of at least 0.3 mcg/kg/min or higher. The sensitivity and specifity for DAO in the anhepatic phase with an optimal cut-off value of greater than 33.4 ng/mL were 80% and 89%, respectively. Results of ROC curve analysis of DAO in the reperfusion period and plasma histamine in the anhepatic phase and after reperfusion were of poor prognostic value to predict higher vasopressor support. In our patient population, the duration of surgery did not significantly correlate with cumulative norepinephrine use.

### Tryptase release and endogenous heparinization

To assess whether changes in histamine concentrations were due to increased mast cell activity occurring during OLT, plasma-tryptase concentrations were quantified. In all except one patient, plasma-tryptase concentrations remained stable after induction of anesthesia (4.3 μg/l, IQR [2.6–6.8] vs. 4.1 μg/l, IQR [2.6–6.3]. In one patient, plasma-tryptase concentrations increased by 82% from baseline, fulfilling the criteria for mast cell activation^21^. In this patient, we measured the highest plasma histamine concentration prior to skin incision (48 nM; i.e. > 3SDs above the median of OLT patients included into this study).

In 4 out of 5 patients undergoing OLT, the anti-FXa activity increased from < 0.1 U/mL at baseline to 0.1 U/mL [0.1–0.75] in the anhepatic phase. In addition, in all patients aPTT increased significantly in comparison to baseline, and delayed clotting was observed in thromboelastometry without heparinase. Taken together, these results suggest endogenous heparinization in patients undergoing OLT.

## Discussion

The present study examined the regulation of histamine and its degrading enzyme DAO in patients undergoing OLT, and assessed whether histamine or DAO correlate with norepinephrine requirements during OLT. Baseline histamine and DAO concentrations were significantly elevated in patients undergoing OLT. Plasma histamine concentrations markedly decreased during OLT, whereas DAO concentrations increased manifold, which correlated with the intraoperative norepinephrine requirements during OLT.

Previous studies have demonstrated that plasma histamine concentrations were greater in cirrhotic patients compared to healthy controls^6,20^. Our findings are in agreement and extend those findings to ESLD patients scheduled for OLT. Greater plasma histamine concentrations in patients with ESLD likely result from reduced hepatic clearance of histamine in advanced liver disease^22^. In the clinical setting of OLT an anhepatic period is required, where the hepatic clearance of histamine is completely abolished. Therefore, it would be expected that histamine plasma concentrations increase during OLT. Surprisingly, plasma histamine concentrations decrease during the anhepatic phase and after reperfusion in patients undergoing OLT when compared to histamine concentrations at baseline. This may partially be due to intravenous administration of 40 mg dexamethasone before the anhepatic phase as part of immunosuppression protocol in our patients. In fact, there is no sufficient literature about effects of steroids on histamine and DAO levels in patients suffering from ESLD or undergoing OLT. Dunsky *et al*. described in their study that corticosteroids induce a prominent decrease in leucocyte histamine due to a depletion of basophils without a decrease in histamine content per basophil in atopic subjects^23^. Another study in newborn rat intestine suggested that DAO activity may be induced by hydrocortisone^24^. Elevated DAO levels observed in our patients, and consequently raised metabolism of histamine may have further decreased plasma histamine concentrations.

In contrast to our results, Lorenz *et al*. demonstrated that plasma histamine concentrations increase in pigs exposed to experimental liver transplantation^25^. However, in contrast to human OLT, no steroids have been administered at any time in this study. Another study showed that systemic histamine concentrations increase in patients undergoing major abdominal surgeries. The increase in histamine concentrations was explained by administration of anesthetic drugs, infusion of plasma and manipulation of the gut^7^. Although similar triggers for histamine release suggested by Lorenz *et al*. were also present during OLT in our patients, the systemic histamine concentration decreased. Some explanations exist for these contradicting results. Firstly, different anesthetics and muscle relaxants, which are not in routine clinical use anymore, were used by Lorenz *et al*. Histamine release by older anesthetics and muscle relaxants was a well-described phenomenon, and could be reduced by the development of newer anesthetics and muscle relaxants such as propofol, sevoflurane and rocuronium, which were used in our study. In addition, the porcine OLTs described by Lorenz *et al*. were performed on porto-jugular bypass^25^, which was not the case in our study. Furthermore, in the study of Lorenz *et al*., abdominal surgeries were performed without caval clamping^7^. Of note is, that physiological alterations largely differ between surgeries performed in bicaval clamping without bypass and those performed with bypass or without bicaval clamping. These varying physiological conditions might serve as an explanation for different histamine secretion patterns between studies.

This is the first systematic investigation demonstrating elevated concentrations of DAO in patients with liver disease. Of note, DAO is rapidly metabolized by rat liver^26^. Thus, the increased baseline concentration of DAO might be explained by a decreased clearance of DAO in patients with ESLD. The most striking finding of this study was the manifold increase in DAO concentrations during OLT comparable in magnitude to early pregnancy^27^, and the likely resultant inverse correlation between DAO and histamine. The increase in systemic concentration of DAO during OLT can explain the degradation of histamine. We have recently shown that plasma levels of DAO (29–52 ng/mL) comparable to those observed in the anhepatic phase can rapidly lower plasma histamine concentrations by ~50–85% within 10 minutes^27^. Thus, the increase in DAO is a mechanistic explanation for the observed decrease in histamine concentrations in patients undergoing OLT.

A decrease in the hepatic clearance of DAO during the anhepatic phase is conceivably a major contributor to the acute and massive rise in DAO. However, several other factors may increase the release of diamine-oxidase during OLT. Heparin is known to release DAO into the systemic circulation^18,28,29^, and it has been described that heparin metabolism is impaired in liver failure^30^. Our results suggest that endogenous heparinization is present in patients undergoing OLT. Thus, the observed increase in systemic DAO concentration during OLT might be explained by the absence of hepatic clearance of heparin during the anhepatic phase^30–32^. Moreover, prothrombin complex concentrates (PCC) contain variable concentrations of heparin^33^, and administration of PCC during OLT could increase systemic heparin concentrations. The elevated heparin concentrations after administration of PCC could trigger the secretion of DAO. However, we found substantial increases in DAO concentrations in patients not receiving PCCs, making an association between administration of PCC and elevated DAO concentrations unlikely. Another pathophysiological pathway contributing to the gradual increase of systemic DAO concentration during OLT might be the DAO release from the gut. Cross clamping of the caval vein leads to a reduction of the cardiac output and subsequently to an intestinal hypoperfusion with venous congestion, representing a period of temporary intestinal ischemia^34^. The following breakdown of the intestinal mucosal barrier is associated with a high DAO release from the intestinal mucosa into the circulation^24,35^. Taken together, the increased DAO concentrations observed in patients undergoing OLT might be a net effect of reduced hepatic DAO clearance, increased heparin induced DAO release and DAO release from the gut injured by transient hypoperfusion during OLT.

Histamine plasma concentrations as low as 11 nM increase the heart rate by approximately 15% and widen the gap between systolic and diastolic pressure by 10% in healthy humans^3^. Experimental studies yield conflicting results when assessing the association between histamine and cardiovascular changes during liver transplantation. While Hansen *et al*. proposed that histamine does not contribute to arterial hypotension after revascularization during OLT in pigs^5^, Lorenz *et al*. demonstrated that administration of antihistaminergic drugs during porcine OLT diminished arterial hypotension in the revascularization phase^25,36^. In the current study, histamine concentrations did not correlate with the norepinephrine requirements in patients undergoing OLT. Uniformly low levels of histamine due to degradation by DAO may have contributed to that finding. In contrast, we found that plasma DAO concentrations correlate with the norepinephrine requirements in the anhepatic phase and after revascularization during OLT. Norepinephrine is used to counteract hypotension which can result in or reflect intestinal hypoxemia, which may itself trigger DAO release. For example, hemorrhagic shock increased DAO levels 6-fold in rabbits^27^, and an hour of intestinal ischemia increased DAO concentrations 3.6-fold in rats^24^. Such an increase in DAO is conceivably accentuated in the presence of decreased clearance of DAO during the anhepatic phase. Interestingly, DAO levels also rise 100-fold in anaphylactic shock in mastocytosis patients^17^, so that increased DAO release levels may partly reflect intestinal ischemia.

The current study has some limitations; the sample size was relatively small and therefore does not allow meaningful subgroup analysis according to the underlying etiology. Detailed correlation analysis between measured histamine and DAO concentrations and hemodynamic variables such as heart rate, mean arterial pressure, systemic vascular resistance or cardiac output could not be determined. Thus, we were unable to assess a contribution of histamine or DAO to compromised hemodynamics in patients undergoing OLT. Furthermore, the concentrations of histamine, DAO and tryptase during OLT could be underestimated due dilution by intraoperative crystalloid and colloid administration. Finally, this study did not directly quantify hepatic histamine clearance, since we have only measured histamine concentrations in the systemic circulation and not in the portal vein or the hepatic artery. Despite these limitations, this is the first study assessing concentrations of histamine and DAO in patients undergoing OLT. Our findings demonstrating an inverse correlation between histamine and DAO and the association between DAO and norepinephrine requirements in patients undergoing OLT are novel, and underline that the balance between histamine and DAO is of utmost importance for hemodynamic stability. Further studies assessing the significance of DAO in the clinical and experimental setting of OLT and other major surgeries should be promoted.

## Conclusion

The results of this study show that patients with ESLD undergoing OLT have elevated baseline plasma concentrations of histamine and its degrading enzyme DAO. During OLT, plasma histamine concentrations markedly decreased, whereas DAO concentrations increased manifold. The increase in diamine oxidase is associated with hemodynamic compromise as measured by norepinephrine requirements in patients undergoing OLT.

## Data Availability

The datasets generated during and/or analyzed during the current study are available from the corresponding author on reasonable request.
